# Encapsulation of a Water Molecule inside C_60_ Fullerene: The Impact of Confinement on Quantum Features

**DOI:** 10.1021/acs.jctc.1c00662

**Published:** 2021-08-23

**Authors:** Orlando Carrillo-Bohórquez, Álvaro Valdés, Rita Prosmiti

**Affiliations:** † Departamento de Física, Universidad Nacional de Colombia, Calle 26, Cra 39, 404 Edificio, Bogotá, Colombia; ‡ Escuela de Física, Universidad Nacional de Colombia, Sede Medellín, A. A 3840 Medellín, Colombia; § Institute of Fundamental Physics (IFF-CSIC), CSIC, Serrano 123, 28006 Madrid, Spain

## Abstract

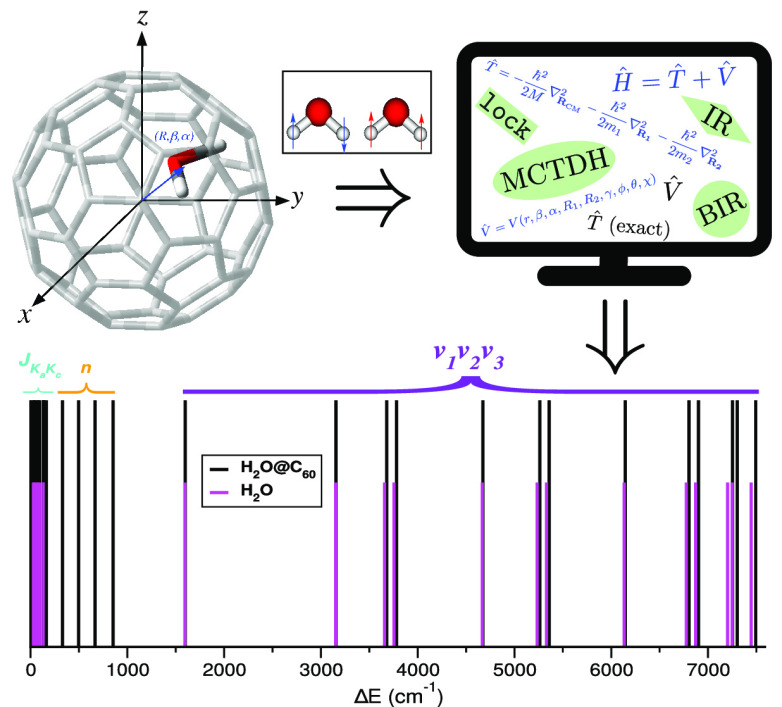

We introduce an efficient quantum fully coupled computational scheme within the multiconfiguration time-dependent Hartree (MCTDH) approach to handle the otherwise extremely costly computations of translational–rotational–vibrational states and energies of light-molecule endofullenes. Quantum calculations on energy levels are reported for a water molecule inside C_60_ fullerene by means of such a systematic approach that includes all nine degrees of freedom of H_2_O@C_60_ and does not consider restrictions above them. The potential energy operator is represented as a sum of natural potentials employing the *n*-mode expansion, along with the exact kinetic energy operator, by introducing a set of Radau internal coordinates for the H_2_O molecule. On the basis of the present rigorous computations, various aspects of the quantized intermolecular dynamics upon confinement of H_2_O@C_60_ are discussed, such as the rotational energy level splitting and the significant frequency shifts of the encapsulated water molecule vibrations. The impact of water encapsulation on quantum features is explored, and insights into the nature of the underlying forces are provided, highlighting the importance of a reliable first-principles description of the guest–host interactions.

## 1 Introduction

Compounds in which atoms or small molecules are trapped in the cavity of fullerenes, such as C_60_ or C_70_, are known as endofullerenes or endohedral fullerenes and offer the opportunity to explore unusual patterns of the entrapped species. The guest confinement inside the fullerene cages influences both the host and the guest, leading to various applications ranging from fundamental research to applied technology in medicine, material science, and electronics, in particular single-molecule transistors for quantum computing.^1−10^ In view of exciting properties and the prospects of applications, as new energy storage materials, among others in nanoscience, these species were revealed to exhibit some unique and unexpected properties, which triggered their extensive investigation from both theoretical and experimental sides.^2,11−31^

A step advance in endofullerene science has been made with the advent of a novel, multistep organic synthesis procedure known as molecular surgery.^9−12,29,32−34^ Such a procedure consists of a series of chemical reactions for creating an open C_60_ cage, in which the guest molecule can be inserted, followed by the closing of the cage with the guest trapped inside. Various molecules, such as H_2_, HD, HF, H_2_O, and CH_4_, have been encapsulated into C_60_ in this manner to date, and their properties have been experimentally investigated by X-ray diffraction, inelastic neutron scattering (INS), far-infrared spectroscopy (FIR), and nuclear magnetic resonance (NMR) spectroscopy techniques.^14,16−18^ Such confined light molecules exhibit dominant quantum effects as a result of strong couplings between translational and rotational motions. Further, H_2_@C_60_, H_2_O@C_60_, and CH_4_@C_60_ endohedral fullerenes are of particular interest, as they also exhibit nuclear spin isomerism, introducing new quantum features, and only certain combinations of nuclear spin and rotational states are allowed by the Pauli exclusion principle.

H_2_O@C_60_ endofullerene, considered as polar C_60_, enclosing a dipolar water molecule in its cage, is particularly intriguing for a variety of reasons. The co-crystallized structure of H_2_O@C_60_ with (NiOEP)_2_ has been determined by single-crystal X-ray diffraction analysis,^12^ and it has been reported^12^ that the oxygen atom of the encapsulated H_2_O molecule is located at the center of the C_60_ cage, with the position of the O–H bonds to be directed toward the Ni ions through the C_60_ cage. Further, on the basis of UV/vis, Fourier transform infrared (FTIR), and NMR spectra analysis of H_2_O@C_60_ and empty C_60_ systems, it has been concluded^12^ that the inclusion of the H_2_O molecule does not affect the structure of the C_60_ cage, indicating no detectable electronic interaction between water and the cage. However, theoretical molecular dynamics (MD) and electronic structure studies^13,35−42^ have shown that there are significant noncovalent interactions between the encaged water molecule and the fullerene cage through formation of two O–H···C hydrogen bonds and various very weak O···C contacts. In particular, the O–H bond lengths of the encapsulated H_2_O have been found to increase compared to isolated H_2_O, affecting both O–H asymmetric and symmetric stretching vibrational frequencies.^38^ In addition, a theoretical investigation on the covalent binding of H_2_O to C_60_ upon compression of H_2_O@C_60_ endofullerene has revealed^15^ that the pressure-induced intercavity chemical reaction can take place depending on the direction of the compression, leading to the formation of endohedral covalent fullerene, if applied in the direction of the two opposite pentagons. In addition, earlier INS, FTIR spectroscopy, and cryogenic NMR experiments^14,18,27,30,43^ and more recent INS spectra^17^ in highly pure samples of solid H_2_O@C_60_ have revealed energy splitting in the ground state of the encapsulated ortho-water, raising the 3-fold degeneracy into single and doublet states (see refs (44) and (26) therein), associated with symmetry breaking of the water environment.

From a theoretical perspective, the investigation of such unexpected properties of water endofullerene requires consideration of quantum treatments in both electronic and nuclear degrees of freedom, and a variety of theoretical models and scenarios have been elaborated^21,22,25,28,45,46^ to date to explore the experimental observations. In view of the complexity of the problem, most of the previous studies^22,44^ have highlighted the importance of fully coupled quantum treatments of both nuclear and electronic degrees of freedom of such nine-dimensional (9D) guest–host systems. Thus, our current investigation^22,47−53^ aims to contribute to the field by proposing a systematic protocol to crosscheck first-principles methodologies on noncovalent guest–host interactions and an efficient procedure within the multiconfiguration time-dependent Hartree (MCTDH) framework^54,55^ to treat the nuclear quantum dynamics of any nanoconfined light molecule. Here, our efforts are focused on fully coupled quantum treatments employing available three-dimensional (3D) and six-dimensional (6D) potentials for the isolated and enclathrated H_2_O molecule, respectively. So, the problem is conveniently discussed in two stages: (a) rotational, translational, and vibrational quantization of a nanoconfined light–heavy–light molecule through a novel computational implementation of an efficient Radau coordinate Hamiltonian representation, allowing to converge the 9D fully coupled computations of highly excited vibrational states of H_2_O@C_60_ and (b) the effect of water molecule encapsulation on its quantum spectral properties due to interfullerene interactions.

The paper is organized as follows. In Section 2, we describe our approach and outline the computational details employed. In Section 3, we present benchmark results on rotational and novel translational and vibrational energy values, as well as comparisons, in cases that are available with previously reported values. Final conclusions and future directions are drawn in Section 4.

## 2 Computational Details

In this study, the methodology already developed in ref (22) is exploited for any light–heavy–light encapsulated molecule to describe the quantum mechanical features of H_2_O@C_60_ endohedral fullerene. We demonstrate the robustness of the method on systems that cannot be described through simple models by carrying out high-accuracy calculations of the molecular levels of the system.

### 2.1 Coordinate System and Hamiltonian Operator

**Figure 1:**
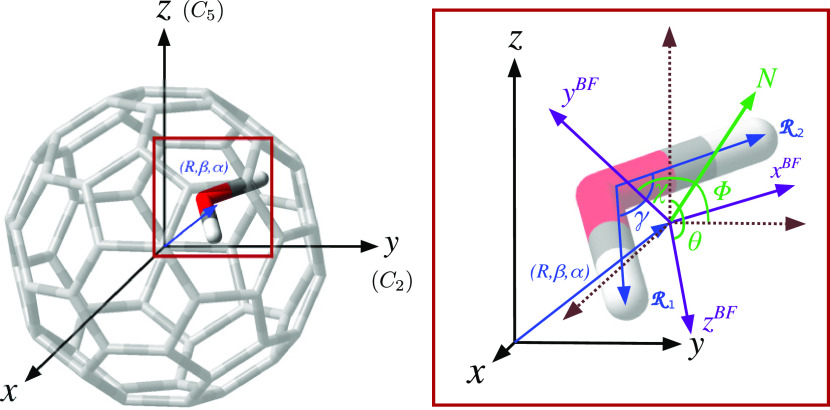
Coordinate systems used in this work to represent a water molecule inside the C_60_ cage. The spherical (*R*, β, α) coordinates describe the center of mass of H_2_O in the space-fixed (SF) coordinate system (see the left-side panel), while the internal Radau (, ,γ) coordinates describe the water molecule in the body-fixed (BF) system, with the three Euler (ϕ, θ, χ) angles connecting the BF and SF (right-side panel) systems.

In Figure 1, we display the 9D coordinate system used to represent the Hamiltonian operator of the fully coupled system, consisting of a flexible H_2_O molecule in rigid C_60_ cages. The origin of the Cartesian *xyz* system is fixed at the center of the fullerene cage with the *z*-axis chosen to lie along the *C*_5_ symmetry axis of C_60_, while the *y*-axis coincides with one of the *C*_2_ symmetry axes. The position of the water molecule’s center of mass inside the fullerene cage is described by means of the spherical coordinates (*R*, β, α) with respect to the *xyz* system, where **R** is the center of the mass vector of the water molecule, and β and α are its polar and azimuthal angles, respectively. The BF coordinate system *x*^BF^*y*^BF^*z*^BF^ refers to the space-fixed *xyz* system through the set of Euler (ϕ, θ, χ) angles (cf. Figure 1), with its origin at the center of mass of the water molecule. An appropriate set of coordinates to describe a triatomic light–heavy–light molecule,^56−58^ as H_2_O, is the internal Radau coordinates (, ,γ), where  and  are the position vectors of the hydrogen atoms with respect to the Radau canonical point,^56^ and γ the angle between them. The *z*^BF^ axis was chosen parallel to the  vector, whereas  lies over the *x*^BF^–*z*^BF^ plane.

The Hamiltonian operator, *Ĥ* = *T̂*(***q***) + *V̂*(***q***), was deduced in the set of  coordinates, with the corresponding exact expression for the kinetic energy operator (KEO) derived as  (see in the Supporting Information (SI) for the extended form, as a particular case of the general formulation in ref (59)), and *V* is the overall potential energy surface (PES). The first term to the right of *T̂* is the operator associated with the movement of the center of mass (translational kinetic energy), and *M* = *m*_O_ + 2*m*_H_ is the total mass of the water molecule, with *m*_O_ = 29 156.95 au and *m*_H_ = 1837.15 au being the oxygen and hydrogen masses, respectively. The advantage of using internal Radau coordinates relies on the lack of crossing terms in the expression of the *T̂* operator, which increases the computational cost at the moment to solve numerically the multidimensional nuclear Schrödinger equation.

**Figure 2:**
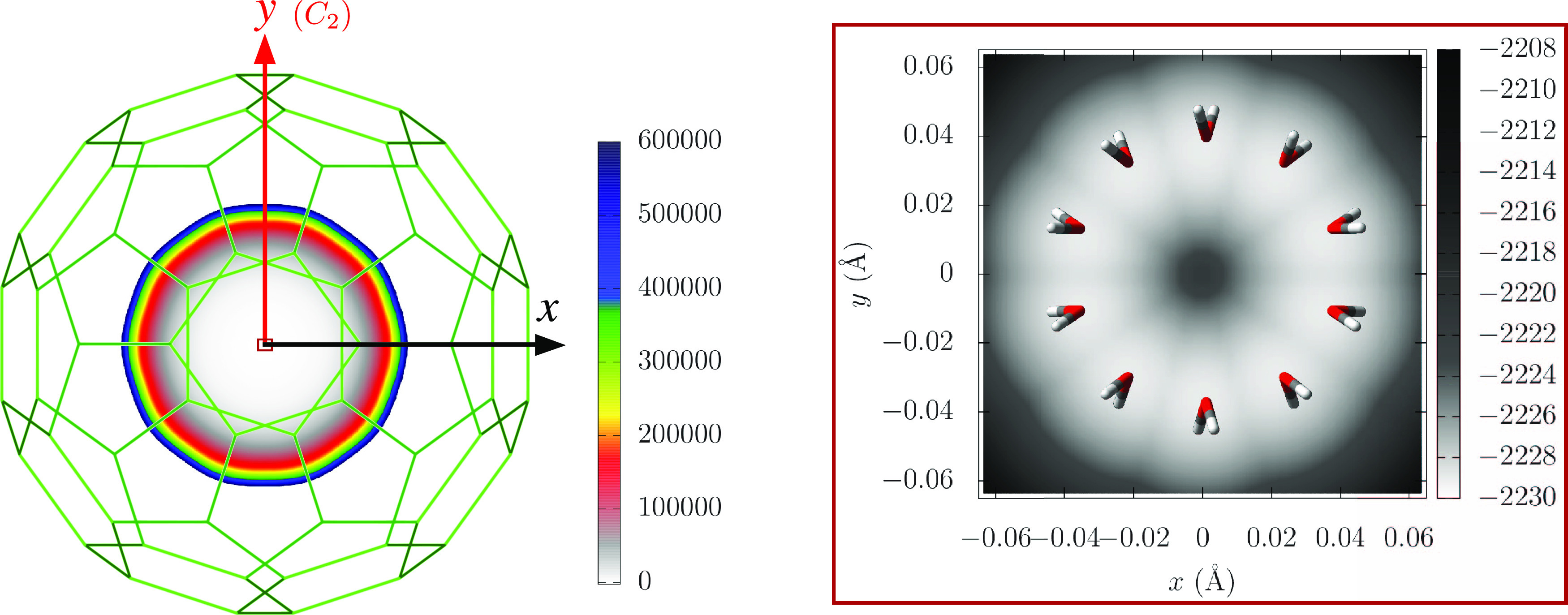
2D contour plot cuts of the *V*_H_2_O–C_60__ potential in the *xy*-plane at *z* = 0, together with the top view of the C_60_ cage (left-side panel). The equivalent 10 minimum energy configurations of the water molecule along the C_2_ axes of the C_60_ cage are also displayed (see the enlarged view in the right-side panel). Equipotential curves are given in cm^–1^.

In previous works of the H_2_O@C_60_ system,^22,44,60^ the potential term, *V*, was built up employing the sum-of-potentials approach, with the intermolecular interaction between the water molecule and C_60_, plus the intramolecular water potential, . The *V*_H_2_O–C_60__ potential was generated as a sum over the H_2_O–C pairwise interactions, modeled with the H–C and O–C Lennard-Jones (LJ) 12-6 potentials adjusted to DFT-SAPT ab initio graphene–water reported in ref (39), while the water monomer potential was taken from ref (61). Figure 2 displays contour plots of the potential in the *xy*-plane at *z* = 0, keeping the water molecule fixed at its equilibrium geometry, while its orientation inside C_60_ is optimized at each grid point. The minimum potential energy value is −2229.79 cm^–1^ at  (0.036,2.12,,0.939,0.939,1.88,5.63,1.42,5.63), with radial coordinates in Å, and angular ones in rad. The C_60_ cage has 10 C_2_-axes, and we should note that each of the symmetric minimum energy configurations is located along them, at , with *n* = 1–10.

### 2.2 MCTDH Computational Setup

Within the MCTDH scheme,^55^ the time-dependent Schrödinger equation is solved by expanding the wave function in a sum of products of the time-dependent low-dimensional basis of so-called single-particle functions (SPFs), ϕ(*Q*, *t*), so Ψ(*q*_1_, ..., *q*_*f*_, *t*) = Ψ(*Q*_1_, ..., *Q*_*p*_, *t*) = ∑_*J*_*A*_*J*_Φ_*J*_, where *f* and *p* are the number of degrees of freedom and the number of particles or combined modes, respectively, while *A*_*J*_ is the MCTDH expansion coefficient, with *J* being the composite index (*j*_1_, ..., *j*_*p*_), and Φ_*J*_ is a Hartree product of SPFs, ϕ(*q*, *t*). The SPFs are in turn represented by linear combinations of time-independent primitive basis functions or discrete variable representation (DVR) grids. The solution of the MCTDH equations of motion requires the computation of the mean-field matrices at every time step. Thus, if the Hamiltonian operator is expressed by a sum of products of monoparticle operators, the so-called product structure, then multidimensional integrals can be solved efficiently, allowing the treatment of high-dimensional problems. The kinetic energy operators are in the sum-of-products form, while potential energy operators must be expanded in the product form representation. Within the MCTDH code,^55^ the POTFIT approach^62^ is the default procedure to transform low-dimensional PESs to the product form, while for treating systems of larger dimensionality, alternatives, such as the *n*-mode representation,^63^ the multigrid POTFIT,^64^ Monte Carlo POTFIT (MCPOTFIT),^65^ or those reported more recently, such as the Monte Carlo canonical polyadic decomposition (MCCPD)^66^ and the rectangular collocation MCTDH (RC-MCTDH)^67^ methods are used.

We used an *n*-mode representation of the potential,^63^ as the grid of at least 10^9^ points required for the 9D potential is too large to be treated with the POTFIT algorithm.^62^ By considering the strong coupling between the 9D coordinates for the H_2_O@C_60_ system, we adopted a 7-mode combination scheme as, *Q*_1_ = *R*, *Q*_2_ = [β, α], *Q*_3_ = θ, *Q*_4_ = [ϕ, χ], , , and *Q*_7_ = γ. The full potential representation has the form *V*_*M*_(*Q*_*i*_) = *V*^(0)^ + ∑_*i*=1_^7^*V*_*i*_^(1)^ (*Q*_*i*_) + ∑_*i*=1,≠*j*_^7^*V*_*ij*_^(2)^ (*Q*_*i*_, *Q*_*j*_)+ ··· +∑_*i*=1,≠*j*,≠···_^7^*V*_*ijk*···_^(7)^(*Q*_*i*_, *Q*_*j*_, *Q*_*k*_,···), with *V*^(0)^ being the reference configuration potential value, *V*^(1)^ the intramode terms, while the remaining *V*^(*n*)^ are the 2- up to 7-mode correlation terms.

As previously discussed, for affordable 9D calculations, we proceeded to truncate the full 7-mode expansion by considering the following *n*-mode selective representations of the *V*^9D^ potential. Thus, *V*^9D^(*Q*_1_, ..., *Q*_7_) = *V*^6D^(*Q*_1_,*Q*_2_,*Q*_3_,*Q*_4_) + Δ*V*^6D_*R*1_^(*Q*_2_,*Q*_3_,*Q*_4_,*Q*_5_) + Δ*V*^6D_*R*2_^(*Q*_2_,*Q*_3_,*Q*_4_,*Q*_6_) + Δ*V*^6D_γ_^(*Q*_2_,*Q*_3_,*Q*_4_,*Q*_7_) takes into account the internal coordinates of the water molecule, as well as the position of its center of mass, with the *V*^6D^(*Q*_1_,*Q*_2_,*Q*_3_,*Q*_4_) = *V*^5D^(*Q*_2_,*Q*_3_,*Q*_4_) + Δ*V*^6D_*R*_^(*Q*_1_,*Q*_2_,*Q*_3_,*Q*_4_) term being the exact potential representation of the 6D system, and Δ*V*^6D_*Qi*_^(*Q*_*i*_,*Q*_2_,*Q*_3_,*Q*_4_) = *V*^6D^(*Q*_*i*_,*Q*_2_,*Q*_3_,*Q*_4_) – *V*^5D^(*Q*_2_,*Q*_3_,*Q*_4_) with *i* = 1, 5, 6, 7 being the corresponding terms in the expansion, keeping fixed at their equilibrium values the independent degrees of freedom at each potential term. According to the *n*-mode representation, the resulting nine-dimensional potential was approximated as a sum of potentials of five and six dimensions, and the POTFIT algorithm,^62^ as implemented in the MCTDH code,^55^ was applied to each of these potential terms to represent them as a sum of operators of one dimension (natural potentials). Some details on the convergence of such approaches are given in Figure S1 and Table S1 (see the SI). In particular. in Table S1, we list the POTFIT parameters (number of natural potentials and the relevant regions of the potential) used for each of the *n*-mode terms together with the corresponding root-mean-square (RMS) errors of the potential fits, while in Figure S1 (see the SI), we show the convergence of the *V*^9D^ expansion as a function of *n*-mode expansion terms over a total number of 10^9^ configurations in the range of each of the nine internal coordinates, as defined in Table S2 (see the SI). One can see that 90% of the configurations have a mean absolute error (MAE) of less than 2 cm^–1^, while for the remaining of them, it does not exceed 4 cm^–1^.

Once the Hamiltonian operator was set up, we should also define the parameters of the grid for its representation. Taking into account the singularities of the KEO, we have carefully chosen the type and range of the DVR basis sets employed in each coordinate. Thus, the underlying primitive basis sets used in the MCTDH calculations are the harmonic oscillator (HO) and radial form solutions (rHO) for radial coordinates, and Legendre (Leg) and exponential (exp) DVR functions for the angular ones depending on the coordinate range, except for the γ angle where the restricted Legendre-type (Leg/R) DVR functions were employed, that allow choosing specific angular range intervals. In Table S2, we list the grid parameters (range, number, and type of primitive basis set functions) for each of the nine coordinates in the MCTDH calculations for obtaining the rotational/translational/vibrational states of H_2_O@C_60_ endofullerene, while Table S3 summarizes the number of SPFs employed in each combination mode in the present computations.

## 3 Results and Discussion

### 3.1 Benchmarking Rotational State Calculations

For calculating rotationally excited states, we employed the block improved relaxation procedure (BIR)^68^ implemented in the Heidelberg MCTDH package.^55^ We initiated with a block of 42 initial vectors corresponding to the number of the desired states that are simultaneously converged collectively, within 10^–3^ cm^–1^, using the same set of SPFs for all of them. This set of 42 eigenstates includes the ground and another 10 (multiple degenerated) lower rotationally excited states. Converging to higher excited states has become more difficult as the excitation energy increases and couplings with translational motions are present.

Thus, here, we assigned pure rotational states, and in Table 1, we show their energy values together with their degeneracy, *g*, and corresponding 2D probability density distributions of one of the degenerated states. Each H_2_O rotational excited state is labeled as *J*_*K*_*a*_*K*_*c*__, according to quantum numbers of total rotational angular momentum *J*, while *K*_*a*_ and *K*_*c*_ are related to the angular momentum for prolate and oblate symmetric tops, respectively. *J*_*K*_*a*_*K*_*c*__ states have a 2*J* + 1 degeneracy and can be further classified into *para*-H_2_O (total nuclear spin *I* = 0, and even parity for *K*_*a*_ + *K*_*c*_) and *ortho*-H_2_O (*I* = 1 and *K*_*a*_ + *K*_*c*_ of odd parity) states. The ground state of *para*-H_2_O is singlet 0_00_, while the ground *ortho*-H_2_O state is triplet 1_01_. We found the ground state energy for H_2_O@C_60_ at *E*_H_2_O@C_60__^0^ = −1967.37 cm^–1^, which agrees very well with the value of −1966.64 cm^–1^ reported recently from quantum calculations^44^ and in our previous work^22^ using a set of internal valence coordinates instead of the Radau grid. Apart from the ground state, small differences are also observed for the rotationally excited levels. Such subtle deviations can be attributed to the differences between the spatial positioning of the grids used in each set of coordinates in the present and earlier 9D MCTDH calculations^22^ or to different molecular parameters and potentials used for the water molecule.^44^

**Table 1:**
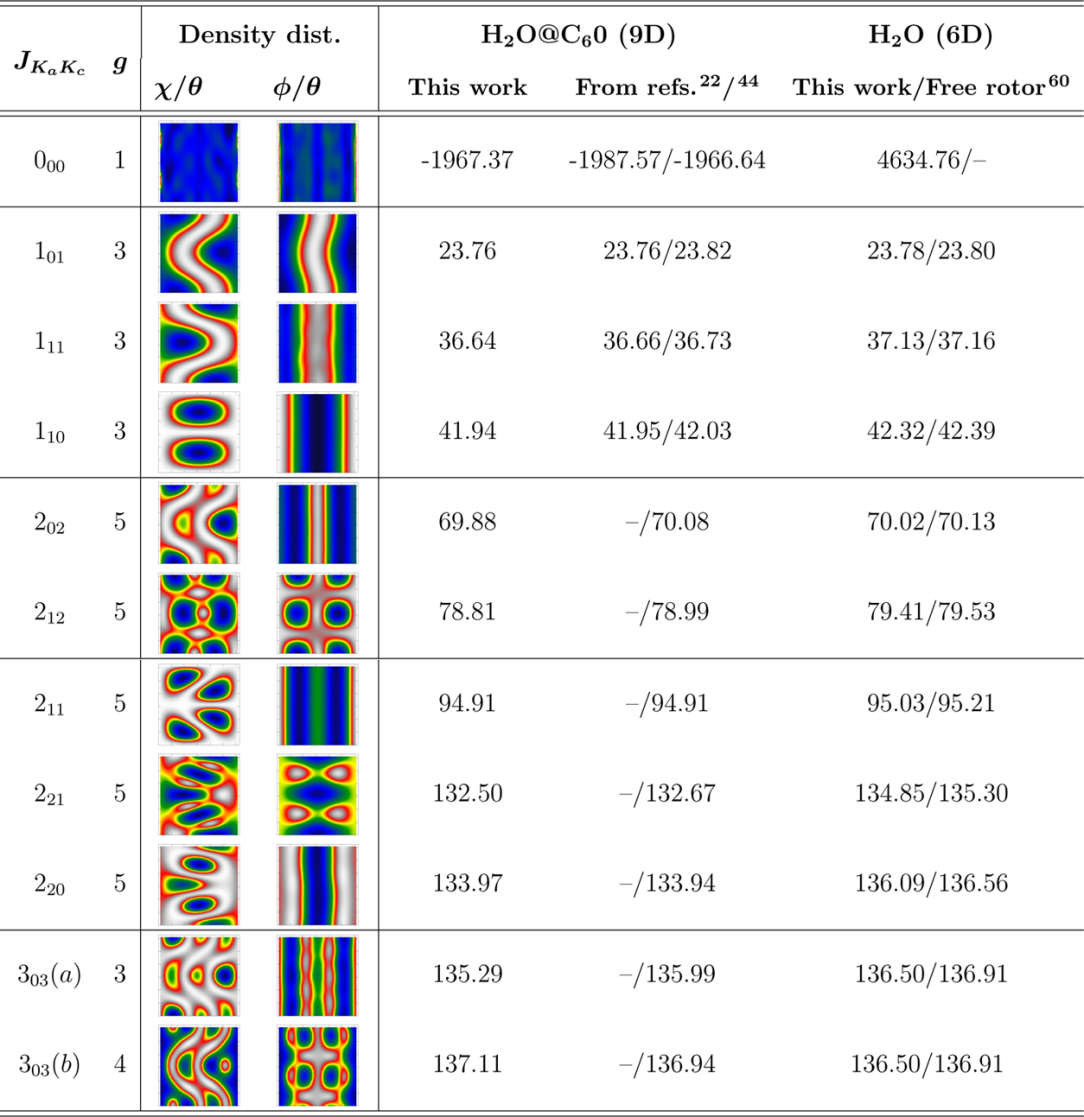
Energies (in cm^–1^) of the lowest rotationally excited states of the H_2_O@C_60_ and H_2_O systems, relative to their ground state energy, *E*_0_, and their assignment, *J*_*K*_*a*_*K*_*c*__, from the present 9D and 6D MCTDH calculations, respectively, where *g* indicates the state’s degeneracy of the encapsulated H_2_O^a^

a Comparison with recently reported values^22,44,60^ is also presented.

**Figure 3:**
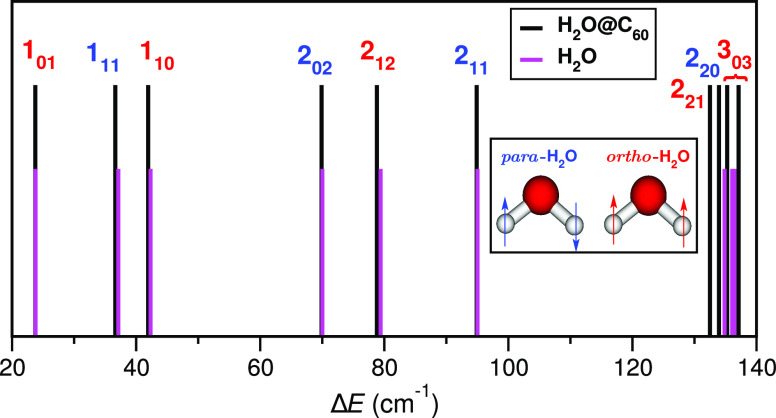
Comparison of the rotational frequencies (see vertical sticks’ positions) computed from the present 9D and 6D MCTDH calculations for the C_60_ encapsulated and isolated H_2_O molecules.

Also, Table 1 shows the energy levels obtained for the isolated H_2_O molecule in the present 6D MCTDH calculations in comparison with those reported in the literature^60^ for a freely rotating rigid water molecule. One can see that the energies of the lower rotational levels of the encapsulated *para*- and *ortho-*H_2_O are close (within about 0.5 cm^–1^) to those of the isolated freely rotating H_2_O, indicating a rather weakly hindered rotation of the trapped molecule. However, as the excitation energy increases, the observed energy differences also increase, with values up to 2.3 cm^–1^ between those of the isolated and encapsulated water molecules, for the higher rotational states in the present work (see Figure 3). In addition, for *J* ≥ 3, rotational levels of the isolated water molecule appear with the 2*J* + 1 degeneracy, while the presence of the icosahedral, *I*_*h*_ symmetry, C_60_ cage, breaks such degeneracy. For example, in the case of *J* = 3, the 7-fold-degenerated 3_03_ levels of the isolated water molecule at an energy of 136.50 cm^–1^ are split into a pair of 3-fold-degenerated, namely 3_03_(*a*), and 4-fold-degenerated, namely 3_03_(*b*), levels at energies of 135.29 and 137.11 cm^–1^, respectively, for the encapsulated H_2_O molecule. The energy splitting of the 3_03_ levels induced by the nonsphericity of C_60_ is 1.8 cm^–1^, indicating a rather small deviation. In the same vein, by comparing the corresponding probability density distributions between the isolated 3_03_ and trapped 3_03_(*a*) water molecule rotational states, we identified some tiny differences, mainly in the ϕ/θ coordinates, which could indicate some preferent orientation of the encapsulated water molecule into the cage.

### 3.2 Novel Translational and Vibrational State Calculations

As earlier discussed, the rotational and translational degrees of freedom of the water molecule inside C_60_ are strongly coupled, with a rigorous approach requiring also their full coupling to the vibrational water modes. However, we should emphasize that intermolecular rotational/translational frequencies are much lower than those of intramolecular vibrational ones; thus, a large number (several hundred) of highly excited rotational–translational states are lying between vibrational excited states, which makes computations rather expensive, and in cases, when the excitation energy increases, even prohibitive. Thus, so far, such issues on fully 9D coupled rotational–translational–vibrational excitations have not been widely addressed by theory, and only a few attempts have been reported in the literature^22,44,47,69,70^ for nanoconfined systems. Indeed, the computationally most challenging objective was to compute specific vibrational states of the water molecule inside the C_60_ cage that allows identifying frequency shifts in H_2_O vibrational modes, indicating further possible observable consequences of its encapsulation.

To perform such calculations of high-lying translational and vibrational states of H_2_O@C_60_, we employed the improved relaxation (IR) method,^71^ as implemented in the Heidelberg MCTDH code.^55^ The energy relaxation method uses imaginary time propagation of the wave function to generate the ground state of a system. The relaxation can be accelerated and, also, excited states can be computed if the MCTDH coefficient vector is not determined by relaxation but by diagonalization. Thus, we choose an initial state that has a reasonable overlap with the eigenstate of interest. Then, the Hamiltonian is diagonalized by a Davidson routine within the SPFs, followed by the selection of the specific eigenstate, the calculations of the mean fields, the relaxation of the SPFs, and so on, until convergence is achieved. For the calculation of the ground state, the lowest energy eigenvector is selected, while for any individual translationally or vibrationally excited state, the eigenvector that has the largest overlap with the initial state is chosen. Converging to excited states is numerically more demanding and depends mainly on how well separated are the states of interest. Then, we can, in principle, converge to any of them by selecting the appropriate initial wave function.

Both translational and vibrational energy levels are computed from the 9D Hamiltonian. A set of lock calculations (whose algorithm is implemented in the MCTDH package^55^) were performed along with the IR method. With the lock keyword, a calculation converges to the eigenvector with the largest overlap with the initial wave function; so, setting a proper set of 60/20 translational-/vibrational-like states, initially obtained from BIR MCTDH calculations with a small and selective basis set of SPFs, it was possible to achieve convergence toward the actual translational or vibrational eigenenergies and eigenstates of each system. Types and numbers of DVR basis functions, together with the mode assignation, can be consulted in Tables S2 and S3, respectively.

**Figure 4:**
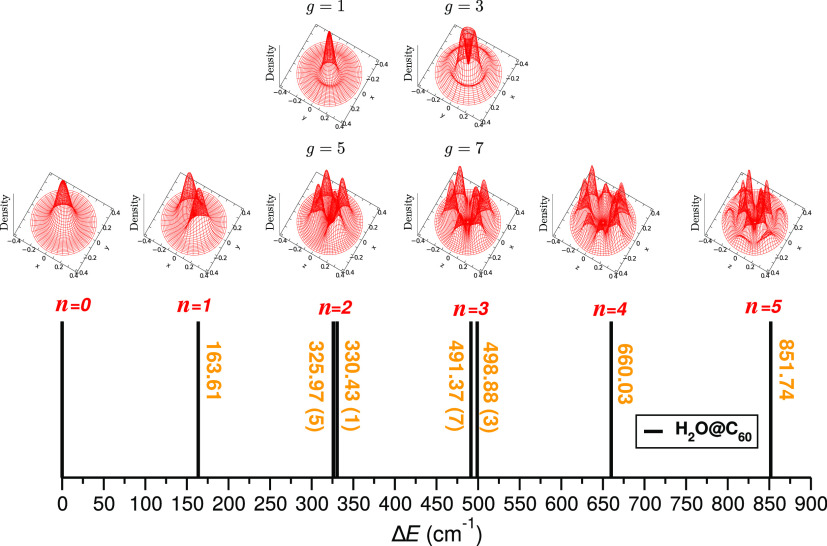
Probability density distributions for the ground and lowest excited translational states. Levels are labeled with the same principal quantum number *n* for the 3D isotropic oscillator. Cartesian coordinates are given in Å and energies in cm^–1^.

Such calculations for the first five pure translationally excited states of H_2_O@C_60_ endofullerene were performed, and their energies with respect to the ground state energy, together with representative probability density distributions in the translational Cartesian coordinates, are plotted in Figure 4. Translationally excited states were assigned according to the principal quantum number *n* = *n*_*x*_ + *n*_*y*_ + *n*_*z*_ of the 3D isotropic harmonic oscillator, and each of them has a *g*_*n*_ = (*n* + 1)(*n* + 2)/2 degeneracy. The first translationally excited state of *para*-H_2_O, labeled as *n* = 1, is a triplet (*g* = 3 with one quantum in each *n*_*x*_/*n*_*y*_/*n*_*z*_ coordinate) state at an energy of 163.61 cm^–1^ above the ground E_0_ state, corresponding to the fundamental translational excitation. This value agrees well with those previously reported at 162.08 and 163.04 cm^–1^ from 6D and 9D quantum calculations,^44,60^ respectively. Higher excited levels with two to five quanta of translational excitation are also computed from the present 9D MCTDH calculations and assigned to *n* = 2 (*g* = 5/1), 3 (*g* = 7/3), 4 (*g* = 9/5/1), and 5 (*g* = 11/7/3) states at energies of 325.97/330.43, 491.37/498.88, 660.03, and 851.74 cm^–1^, respectively. The energy splitting found between the degenerate translationally excited levels, e.g., 4.5 and 7.5 cm^–1^ in the *n* = 2 and 3 levels, respectively, reflects the anharmonicity in this mode. In a previous study,^60^ values have been reported only for the second *n* = 2 excited states from a 6D rigid rotor model, corresponding to *l* = 2 and 0 at energies of 325.97 (*g* = 5) and 328.21 (*g* = 1) cm^–1^, respectively. One can see that our results are in accordance with these values, while the small differences can mainly be attributed to the fully coupled 9D treatment developed in the present study.

As before, a set of lock IR MCTDH calculations were performed to calculate the vibrational energy levels of H_2_O@C_60_ using the 9D Hamiltonian. Types and numbers of DVR basis functions, together with the mode assignation, can be consulted in Tables S2 and S3 (see the SI). The computed vibrational energies for the encapsulated water molecule in the C_60_ cavity are listed in Table 2, together with the corresponding probability density distributions in (,γ) and (, ) planes. One can see that convergence was achieved for specific vibrationally excited states of H_2_O@C_60_ endofullerene; in total, 13 levels were computed up to energies of about 7500 cm^–1^ above the ground state energy. In turn, on the basis of their probability distributions, each state was assigned as ν_1_ν_2_ν_3_, using the vibrational quantum numbers of the isolated water molecule, with ν_1_ and ν_3_ corresponding to the symmetric and asymmetric OH stretching modes, respectively, while ν_2_ to the HOH bending one. Thus, apart from the fundamentals, we were also able to assign vibrationally excited levels with up to 4 quanta in the bending mode, as well as several combinations with stretching modes. In addition, in Table 2, we also present vibrational energies, obtained here from 3D MCTDH calculations for an isolated water molecule. We found that such 3D results are in accordance with the values reported previously using the same H_2_O PES.^61,72^ Just recently, quantum full 9D calculations have been reported^44^ for the lower four vibrationally excited states of H_2_O@C_60_. We found that the present values agree very well with those energies, within less than 1 cm^–1^.

**Table 2:**
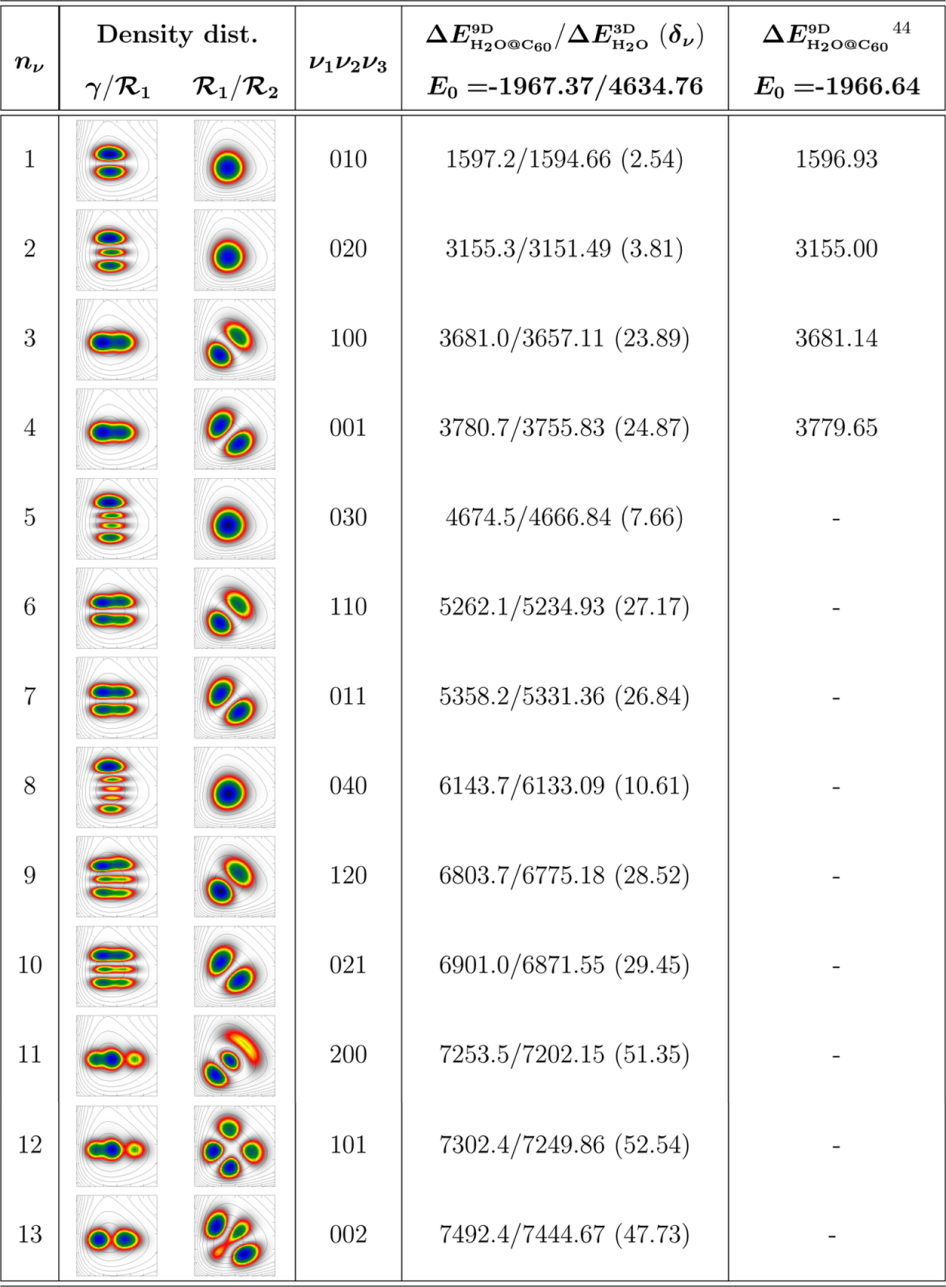
Energies of Vibrationally Excited States (in cm^–1^) for the Encapsulated H_2_O Molecule inside the C_60_ Cage Obtained in the 9D MCTDH Calculations, Relative to the Ground State Energy, *E*_0_^a^

a The corresponding values obtained for an isolated water molecule from 3D MCTDH calculations are also presented for comparison purposes, as well as the frequency shifts, δ_ν_, between the encapsulated and isolated water vibration modes. Plots of probability density distributions, in Radau coordinates, are shown, with  bond lengths in the range of 0.7–1.3 Å and angle γ in 0.9–3.0 rad.

**Figure 5:**
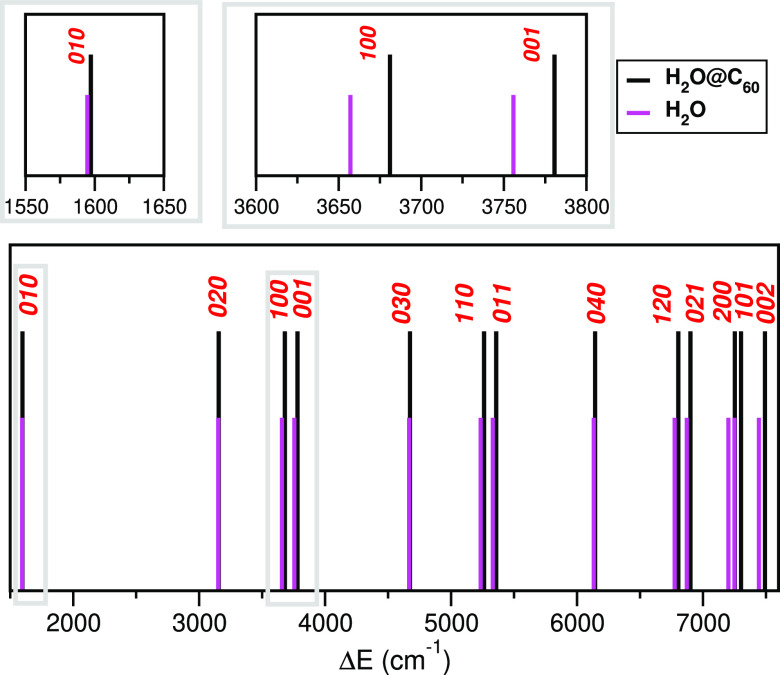
Comparison of the vibrational ν_1_ν_2_ν_3_ frequencies (see vertical sticks’ positions) computed from the present 9D and 3D MCTDH calculations for the C_60_ encapsulated and isolated H_2_O molecules.

By comparing now our energy values from the 9D and 3D calculations (see Figure 5) for the encapsulated and isolated water molecules, respectively, we found that both ν_1_ and ν_3_ OH vibrational fundamental frequencies and overtones of the H_2_O molecule undergo appreciable upshifts upon its encapsulation, while the ν_2_ bending mode exhibits a much smaller one. In particular, OH stretching fundamentals of the C_60_-encapsulated H_2_O molecule are blue-shifted from those of the isolated gas-phase water molecule by 24 and 25 cm^–1^, while the 010 HOH bending fundamental and its 020 overtone are blue-shifted by only 2.5 and 3.8 cm^–1^, respectively, much less than the stretching ones. The latter values are in accordance with previously reported values of 1–6 cm^–1^ from PBE0/6-311++G(d,p) harmonic frequency optimization calculations^38^ and 2.19 and 3.38 cm^–1^ from the quantum 9D calculations by Felker and Bačić.^44^

So far, frequency blue shifts of the stretching H_2_O modes have been reported by different theoretical treatments.^35,36,38,39,44^ In particular, normal mode analysis from Hartree–Fock optimizations^35^ has predicted blue shifts of 41 and 30 cm^–1^ for the symmetric and asymmetric H_2_O stretch modes, respectively, while red shift values of 24–35 and 39–51 cm^–1^ reported from more recent PBE0/6-311++G(d,p) calculations^38^ for various least energy H_2_O@C_60_ conformers, which are smaller for the symmetric mode than the asymmetric mode. Further, instantaneous vibrational analysis applied to molecular dynamics simulation configurations at the B3LYP/cc-pVDZ(STO-3G) level of theory has reported blue shifts of 13–19 cm^–1^ only for the symmetric stretching mode, whereas no shift has been found for the asymmetric one.^36^ Finally, more recently, OH bond vibrational frequency blue shifts from the gas-phase water, of similar values of 14 and 15 cm^–1^ for the symmetric and asymmetric OH stretching modes, respectively, have been reported^39^ from classical MD simulations using the same DFT-SAPT force-field parameters^39^ as those in the present work.

Our estimated blue shift values are compared quite well with those given by Farimani et al.^39^ and are very close (within 1 cm^–1^) to the values of 24.2 and 23.8 cm^–1^ reported by Felker and Bačić.^44^ However, we should note that in the absence of any experimental evidence on such effects upon water molecule’s encapsulation into the C_60_ cage, together with the contradictory results on the values of the vibrational frequency shifts, one should consider to carefully evaluate the potential models employed in the present and most recent studies, on the basis of first-principles guest–host interactions taking into account many-body contributions.

## 4 Summary and Conclusions

The effect of nanoconfinement on the quantum features of the encapsulated molecules is still an open topic, with relevance in various molecular processes in several scientific areas. In an effort to contribute to the field, a light endohedral fullerene, such as H_2_O@C_60_, was modeled by means of an exact full 9D Hamiltonian, deduced for a system of internal Radau coordinates to describe the encapsulated water molecule, which correlates all of the degrees of freedom and considers anisotropies in the rigid C_60_ cage and the extended H_2_O molecule interaction. One specific focus of the present study was to develop a systematic and computationally efficient procedure to treat quantum mechanically such a highly coupled system within the MCTDH framework. Thus, we have shown that such a quantum computational scheme could be utilized for rigorous and tractable computations of any light–heavy–light encapsulated molecule. The calculations yield well-converged intermolecular low-lying rotational and translational states, as well as intramolecular vibrational fundamentals and overtones up to higher energies of 7500 cm^–1^ of the H_2_O@C_60_ system, and the obtained results for the encapsulated water molecule were compared with those for the isolated molecule.

The results reported can be established as the first reference values in the study of the quantum features of such an endofullerene. In some cases, we have verified, while in others, we have managed to expand the previously reported values in higher energies. We have found small energy splitting in higher rotational levels of the encapsulated water molecule that reflects the nonsphericity of the C_60_ cage, while such splitting in translationally excited levels was attributed to anharmonicities in this mode. Finally, we have predicted significant blue shifts in the vibrational frequencies of the encapsulated water with respect to those of the isolated gas-phase molecule, as an important consequence of its encapsulation in the C_60_ cage.

However, we do realize that conclusions in the present study, as well as in previous ones, are constrained by the limitation of the sum-of-potentials PES used. Although the results are numerically exact for the PES employed, there is evidence from electronic structure calculations of significant noncovalent interactions between the water molecule and the C_60_ cage. Importantly, the lack of such many-body contributions could introduce considerable uncertainty for the PES’s quality. To date, there is no ab initio/first-principles-based interaction potential for such endofullerenes. Thus, our efforts should be directed on the accurate description of such guest–host interactions, through well-converged reference training data sets from wave function and/or reliable DFT approaches, for developing predictive data-driven potential/force-field models in a general automated fashion by sampling the most representative and diverse set of configurations. Following current challenges in the field, in the near future, we aspire to address by proper machine-learned modeling these noncovalent interactions and employ such improved (high-quality) models to carry on further molecular computer simulations, providing decisive results on fully coupled intramolecular and intermolecular excitations and conclusive insights into the impact of the guest–host forces on the quantized dynamics of the encapsulated water molecule.

## Supporting Information Available

Kinetic energy operator in the 9D coordinates; details on the convergence of the *n*-mode potential expansion and the multidimensional POTFIT approach (Figure S1 and Table S1); and specific computational details for carrying out the MCTDH calculations (Tables S2 and S3) (PDF)
