# Supplementary material for: Encapsulation of a Water Molecule inside C60 Fullerene: The Impact of Confinement on Quantum Features
Source: J Chem Theory Comput. 2021 Aug 23;17(9):5839–48. doi: 10.1021/acs.jctc.1c00662 (PMC8444341; doi:10.1021/acs.jctc.1c00662)
Supplement: Supplementary file 1 — ct1c00662_si_001.pdf [file ct1c00662_si_001.pdf]

Encapsulation of water molecule inside C_{60} fullerene: the impact of confinement on quantum features

Orlando Carrillo-Bohórquez[‡,§], Álvaro Valdés[†,*] and Rita Prosmiti[§*]

[‡]*Departamento de Física, Universidad Nacional de Colombia, Calle 26, Cra 39, Edificio 404, Bogotá, Colombia*

[†]*Escuela de Física, Universidad Nacional de Colombia, Sede Medellín, A.A 3840, Medellín, Colombia*

[§]*Institute of Fundamental Physics (IFF-CSIC), CSIC, Serrano 123, 28006 Madrid, Spain*

E-mail: avaldesl@unal.edu.co; rita@iff.csic.es

Supporting Information Available

Kinetic energy operator in the 9D coordinates:

$$
\begin{aligned}
\hat{T} = &-\frac{\hbar^2}{2MR^2}\left[R^2\frac{\partial^2}{\partial R^2} + \cot(\beta)\frac{\partial}{\partial\beta} + \frac{\partial^2}{\partial\beta^2} + \csc^2(\beta)\frac{\partial^2}{\partial\alpha^2}\right] \\
&-\frac{\hbar^2}{2m_{\mathrm{H}}\mathcal{R}_1^2}\left[\mathcal{R}_1^2\frac{\partial^2}{\partial\mathcal{R}_1^2} + \cot(\gamma)\frac{\partial}{\partial\gamma} + \frac{\partial^2}{\partial\gamma^2} + \cot(\theta)\frac{\partial}{\partial\theta} + \frac{\partial^2}{\partial\theta^2} + \csc^2(\theta)\frac{\partial^2}{\partial\phi^2}\right. \\
&\quad + \left(\cot^2(\gamma) + 2\cos(\chi)\cot(\gamma)\cot(\theta) + \cot^2(\theta)\right)\frac{\partial^2}{\partial\chi^2} + 2\cot(\theta)\sin(\chi)\frac{\partial^2}{\partial\gamma\partial\chi} \\
&\quad - 2\csc(\theta)\left(\cos(\chi)\cot(\gamma) + \cot(\theta)\right)\frac{\partial^2}{\partial\phi\partial\chi} - 2\csc(\theta)\sin(\chi)\frac{\partial^2}{\partial\phi\partial\gamma} \\
&\quad \left. +2\cot(\gamma)\sin(\chi)\frac{\partial^2}{\partial\theta\partial\chi} - 2\cos(\chi)\frac{\partial^2}{\partial\theta\partial\gamma}\right] \\
&-\frac{\hbar^2}{2m_{\mathrm{H}}\mathcal{R}_2^2}\left[\mathcal{R}_2^2\frac{\partial^2}{\partial\mathcal{R}_2^2} + \cot(\gamma)\frac{\partial}{\partial\gamma} + \frac{\partial^2}{\partial\gamma^2} + \csc^2(\gamma)\frac{\partial^2}{\partial\chi^2}\right].
\end{aligned}
$$

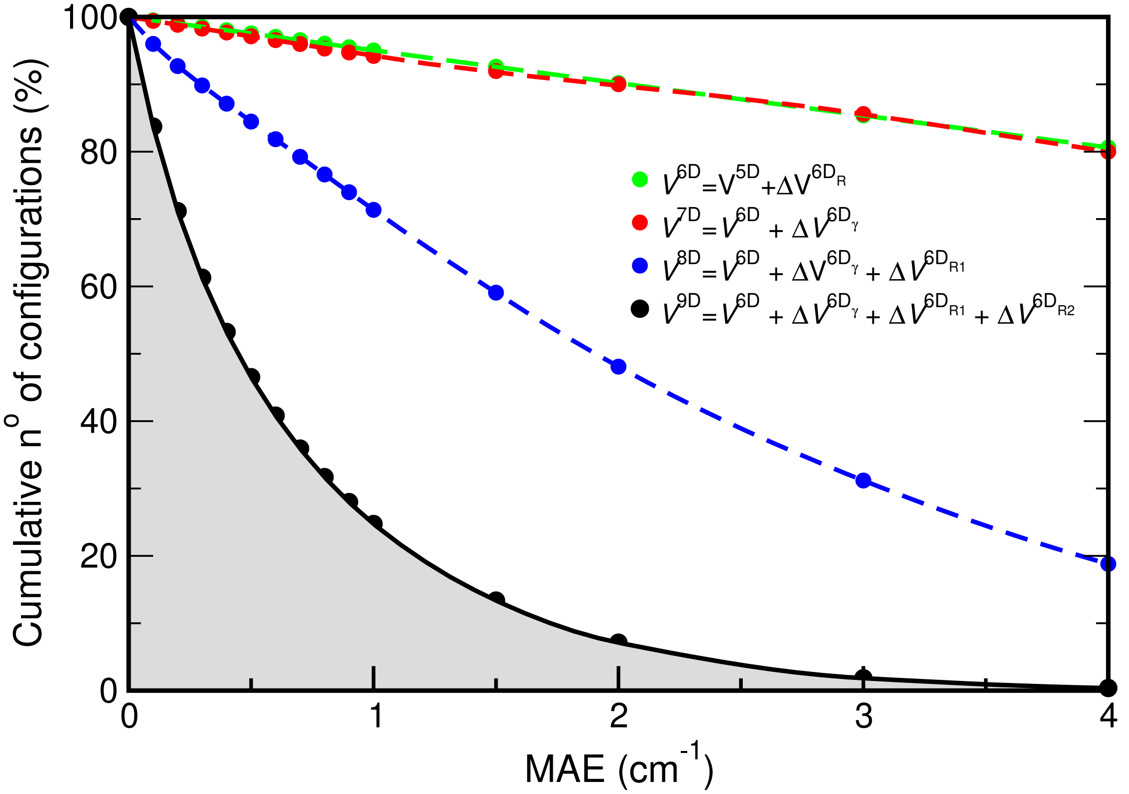

Figure S1:Convergence of n-mode PES representation as a function of the indicated expansion terms (see text).

Table S1: POTFIT parameters used to generate sum-of-products form of each n-mode expansion term of the PES. The number of natural potentials, the relevant regions of the potential together with the root-mean-square (RMS) error for the fits are also given. Contr stands for the mode over which a contraction is done.

Natural potentials	V^{5D}	ΔV^{6D_γ}	$\Delta V^{6D_{R_1}}$	$\Delta V^{6D_{R_2}}$	ΔV^{6D_R}
$N_{\gamma/R_1/R_2/R}$	-	10/-/-/-	-/10/-/-	-/-/10/-	-/-/-/10
N_θ	Contr	Contr	Contr	Contr	Contr
$N_{\phi,\chi}$	25	25	25	25	25
$N_{\beta,\alpha}$	15	15	15	15	15
**Relevant regions**	$V < 2000$ cm^{-1}				
**Min/Max PES(cm^{-1})**	-2229.1/-2191.3	-26.2/26.2	-42.7/306.5	-42.7/306.5	-18.5/1273.1
**RMS error on relevant grid points (cm^{-1})**	0.1	0.2	< 0.02	0.3	1.8

Table S2: Primitive DVR basis sets used in the MCTDH calculation of rotational/translational/vibrational levels for the $H_2O@C_{60}$ system, where the number of employed basis functions and the range of each degree of freedom is specified. Radial coordinates in Å, and angular ones in rad.

**Coordinate**	**DVR basis**	**N^o**	**Grid range**
R	rHO	25/35/25	(0.0,0.38)
β	Leg	35/35/25	(0,π)
α	exp	35/35/25	(0,2π)
$\mathcal{R}_1$	HO	25/25/35	(0.65/0.65/0.61,1.27/1.27/1.45)
$\mathcal{R}_2$	HO	25/25/35	(0.65/0.65/0.61,1.27/1.27/1.45)
γ	Leg/R	25/25/35	(1.26/1.26/0.82,2.65/2.65/π)
ϕ	exp	35/33/25	(0,2π)
θ	Leg	35/33/25	(0,π)
χ	exp	35/33/25	(0,2π)

Table S3: Mode-combination scheme and number of employed SPFs in the indicated ground, rotational, translational and vibrational states IR/IR$_{lock}$ or BIR MCTDH calculations (see text) of the $H_2O@C_{60}$ endofullerene.

**N^o SPFs/Mode**	$\boldsymbol{N_R}$	$\boldsymbol{N_{\beta,\alpha}}$	$\boldsymbol{N_{\mathcal{R}_1}}$	$\boldsymbol{N_{\mathcal{R}_2}}$	$\boldsymbol{N_\gamma}$	$\boldsymbol{N_\theta}$	$\boldsymbol{N_{\chi,\phi}}$
Ground (IR)	5	20	3	3	5	15	70
R (BIR)	4	12	3	3	3	15	60
T (IR$_{lock}$)	7	50	6	6	4	4	6
V (IR$_{lock}$)	2	4	50	50	7	4	6
